# Changes in Primary HIV-1 Drug Resistance Due to War Migration from Eastern Europe

**DOI:** 10.1007/s10903-023-01559-1

**Published:** 2023-11-16

**Authors:** Andrzej Załęski, Agnieszka Lembas, Tomasz Dyda, Ewa Siwak, Joanna Osińska, Magdalena Suchacz, Justyna Stempkowska-Rejek, Marta Strycharz, Justyna Orzechowska, Alicja Wiercińska-Drapało

**Affiliations:** 1 Hospital for Infectious Diseases in Warsaw, Warsaw, Poland; 2 Department of Infectious Diseases, Tropical Diseases and Hepatology, Medical University of Warsaw, Warsaw, Poland; 3 Molecular Diagnostics Laboratory, Hospital for Infectious Diseases in Warsaw, Warsaw, Poland; 4 HIV Out-Patient Clinic, Hospital for Infectious Diseases in Warsaw, Warsaw, Poland; 5 Infectious Diseases Clinical Ward in Ostróda, Department of Family Medicine and Infectious Diseases, University of Warmia and Mazury in Olsztyn, Olsztyn, Poland; 6 Department of Infectious Diseases and Hepatology, Medical University of Lublin, Lublin, Poland; 7 Clinical Department of Infectious Diseases and Hepatology, Medical University of Lodz, Lodz, Poland; 8 Clinical Department of Infectious Diseases, College of Medical Sciences, Medical Center in Łańcut, University of Rzeszów, Rzeszów, Poland

**Keywords:** HIV, ART, Epidemiology, War, Migrants, Ukraine

## Abstract

In recent years, especially as a result of war in Ukraine, enormous movements of migration to Poland from eastern European countries have been reported, including people living with Human Immunodeficiency Virus (HIV). We have conducted multi-center, prospective study, which aimed to establish HIV-1 subtype and assess the presence of primary drug resistance mutations to nucleoside reverse transcriptase inhibitors, non-nucleoside reverse transcriptase inhibitors and protease inhibitors in antiretroviral treatment naïve patients. The clinical trial recruited 117 individuals during 2 years period (2020–2022). The prevalence of HIV-1 subtype A was statistically significantly more frequent in Ukrainian, and HIV-1 subtype B in Polish patients (p < 0.05). Drug resistance mutations were detected in 44% of all cases and the comparison of presence of mutations in the analyzed groups, as well as in the subgroups of subtype A and B HIV-1 has not revealed any significant differences (p > 0.05), nevertheless Polish patients had multidrug resistance mutations more frequent (p < 0.05). The results from our trial show no increased risk of transmission of multidrug resistant HIV strains in our cohort of Ukrainian migrants.

*Clinical trials. Gov number* NCT04636736; date of registration: November 19, 2020.

## Background

Due to economic and political situation, especially as a result of war in Ukraine in 2022, enormous movements of migration to Poland from eastern European countries have been reported in the recent years. According to the Polish governmental statistics, till third quarter of 2022, almost 1.5 million of war refugees from Ukraine have been registered for temporary medical care within national protection schemes [1, 2].

Recently, also substantial increases in diagnosis of human immunodeficiency virus (HIV) infected people have been reported in some countries, including Poland. At the same time 42% of those diagnosed in the European Union/European Economic Area (EU/EEA) in 2018 were migrants, defined as originating from outside of the country in which they were diagnosed. More than one-third (40%) of newly diagnosed cases due to heterosexual transmission were among migrants originating from countries with generalized HIV epidemics, such as Ukraine [3]. Before the war, in Poland almost 27 thousands of people living with HIV (PLWH) were registered, with approximately 15.5 thousands being on antiretroviral treatment (ART) [4]. In the same time Ukraine had 260 thousands of PLWH, with merely the half of them being on ART. Till November 2022, according to the polish national data, 2647 Ukrainian migrants were registered to enter HIV care, what constituted 15% increase in the total treated HIV cases [4–6].

Regardless of the remarkable success in the treatment of HIV-1 infection, there is increasing concern about the emergence of HIV drug resistance mutations (DRMs), which can lead to treatment failure [7]. HIV drug resistance can be transmitted (primary resistance) in previously untreated persons; the resistance can also be acquired when the patient is already on ART [7–9]. Although both, acquired and transmitted HIV drug resistance are public health concerns, transmitted resistance has the potential to reverse the effectiveness of first-line ART more rapidly at the population level [7, 8]. Patients with transmitted drug resistance begin ART with a lower genetic barrier to resistance, a higher risk of virologic failure and a higher risk of developing resistance, even to those drugs in their regimen that were originally fully active [7, 10–13]. Worldwide, the prevalence of DRMs in HIV-1 range from 5 to 25% [9, 13–15].

## Theoretical/Conceptual Framework

**Fig. 1:**
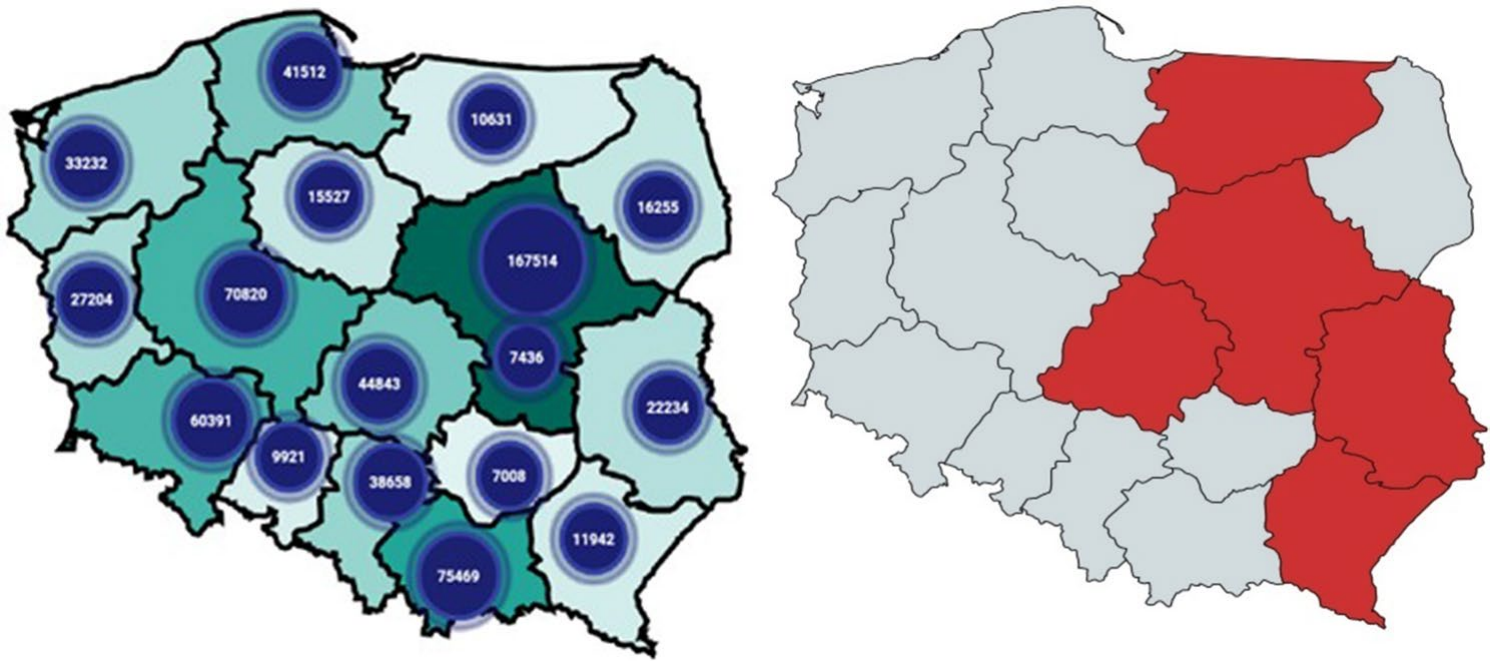
Migration movements and site of recruitment

Surveillance of primary resistance can supply information to support the rational use of ART in prophylactic and therapeutic schemes [16]. Due to changes in epidemiological pattern in newly diagnosed patients with HIV infection in Poland and increase in number of HIV positive patients from Ukraine we aimed to assess whether in newly diagnosed, ART naïve patients the presence of transmitted DRMs in the population of migrants from eastern Europe differs from the pattern of primary resistance in not foreign born individuals (Fig. 1).

## Methods

We have conducted multi-center, prospective, observational, cross sectional study. The patients were recruited during 2 years period (2020–2022). The site of the recruitment of the cohorts were six clinical centers in Poland (two centers in Warsaw and one center in each city: Olsztyn, Lublin, Łódź and Rzeszów), all in the closest regions to the Polish–Ukrainian border, which cover territorially 40% of refugees [1, 2]. The clinical trial received a positive opinion from the Bioethics Committee at the Medical University of Warsaw (Number of consent: AKBE/206/2020) and was registered on clinicaltrials.gov (Number: NCT04636736).

Inclusion criteria were recent diagnosis with HIV-1 infection (newly recognized patients), no history of ART, age 18 years or older. The patients were selected by cooperating physician in collaborating centre on the basis of the polish or foreign nationality when they met the inclusion criteria. We collected epidemiological data such as: age, gender, BMI, origin, migration data, sexual preferences and clinical data: HIV viral load, CD4 + cells count, presence of AIDS-defining diseases, implemented ART, serology of HBV and HCV co-infections. All patients who had positive test result for any of the following: HBs-Ag, anti-Hbc, HBV-DNA were considered individuals with HBV co-infection and all patients who had positive test result for anti-HCV or HCV-RNA were considered HCV co-infected. In all patients, before starting ART, the presence of HIV-1 DRMs to nucleoside reverse transcriptase inhibitors (NRTI), non-nucleoside reverse transcriptase inhibitors (NNRTI) and protease inhibitors (PI). Undetectable viral load was defined as a < 50 copies of HIV-RNA/ml of the peripheral blood and virological failure was defined as a detectable HIV-1 viral load after 6 months from the initiation of the ART.

The drug resistance genotyping of HIV-1 protease (PR) and reverse transcriptase (RT) coding region was performed based on a Sanger sequencing method with use of commercially available ViroSeq HIV-1 Genotyping System (Abbott Molecular, Des Plains, IL, USA). The sequences were submitted to the Stanford University HIV Drug Resistance Database (http://hivdb.stanford.edu) in order to comparison of potential differences in the interpretation of the significance of genetic variants detected. For HIV-1 subtype and circular recombinant forms (CRFs) determination of partial pol submitted sequences REGA HIV-1 Subtyping Tool Version 3.0 was used. The HIV-1 viral loads (VL) were measured by real time PCR system (Abbott). Immunophenotyping and count of T lymphocyte CD4 + and CD8 + subsets were determined using flow cytometry method with application of TriTEST fluorescence-conjugated monoclonal antibodies (BD Biosciences, Australia). Data were acquired and analysed using multichannel dual-laser BD FACSCalibur analyser with BD MultiSET software.

For the statistical evaluation the Shapiro–Wilk test was performed for the verification of the normality of the distributions in the analyzed variables. Student’s t-test or Mann–Whitney U test was used to evaluate the difference in mean value in continuous variables and χ 2 or Fisher exact tests was performed for categorical variables. A p value of < 0.05 was considered statistically significant. Statistical analyses were performed using Python 3.7 software and Statistica 13.1 program (StatSoft Poland, Kraków, Poland).

## Results

### Study Group

The inclusion and exclusion criteria met 117 patients. We have recruited 78 patients from two centers in Warsaw, 28 patients from Olsztyn, 6 from Lublin, 3 from Łódź and 2 patients from Łańcut center. Because of the lack of the results of genotyping in 34 individuals they were excluded from the analysis. In the study group there were 61 patients (52.14%) from eastern countries (34 men, 27 women) and 56 patients (47.86%) from Poland (52 men, 4 women). Among patients from eastern countries there were 58 patients (95.08%) from Ukraine (11 war refugees and 47 patients who arrived to Poland before the onset of war), 3 patients (4.92%) from: Belarus, Georgia and Kazakhstan. The baseline characteristics of the patients are presented in Table 1.

**Table 1:** Study group characteristics

Characteristics of the patients	Patients from eastern countries (n = 61)	Polish patients (n = 56)	P
Baseline characteristics			
HIV viral load (copies/mL) mean (min.-max.)	249095.34 (249–3357752)	672660.43 (304–10000000)	**0.001**
HIV viral load (log_10_ copies/mL) mean (min.-max.)	4.42 (2.40–6.53)	5.07 (2.48–7.00)	**0.001**
Baseline CD4 count (cells/μl) mean (min.-max.)	295.11 (4–922)	217.88 (5–1132)	**0.016**
Route of HIV transmission			
MSM (n/%)	22 (30.07)	40 (71.43)	**0.022**
Heterosexual (n/%)	32 (52.46)	15 (26.79)	**0.013**
IDU (n/%)	5 (8.20)	1 (1.79)	0.209
Iatrogenic (n/%)	2 (3.28)	0 (0.00)	0.497
Comorbidities			
AIDS-defining diseases (n/%)	16 (26.23)	28 (50.00)	**0.047**
HBV coinfection (n/%)	14 (22.95)	4 (7.14)	**0.007**
HCV coinfection (n/%)	11 (18.03)	6 (10.71)	0.225

Bold values indicate statistical significance (p < 0.05)

We observed statistically significant difference in HIV viral load, baseline CD4 cell count, the route of HIV transmission, the presence of AIDS-defining diseases and HBV coinfection in patients from eastern countries in comparison to Polish patients. Eastern countries patients had significantly lower HIV mean viral load at diagnosis: 2,45 × 10^5^ vs 6,73 × 10^5^ copies/ml (4.42 log_10_ vs 5.07 log_10_) (p = 0,001), higher baseline lymphocyte CD4 count: 295 vs 218 cells/µl (p = 0,016), less AIDS-defining diseases: 16 vs 28 (p = 0,047) and more frequently HBV coinfection: 14 vs 4 (p = 0,007). There were less individuals from eastern countries who were infected with HIV via male to male sexual contact (MSM) and more infected via heterosexual contact. In the analyzed group of patients there were 44 people (37.61%) with AIDS-defining diseases, among them 20 individuals (45.45%) had more than one AIDS defining disease at the time of diagnosis. Among AIDS-defining diseases occurring in patients from eastern countries there were significantly more tuberculosis, whereas wasting syndrome was more prevalent among patients from Poland (p = 0.05).

### HIV-1 Subtype

We examined the HIV-1 subtype in the group of Ukrainian and Polish patients. Table 2 shows the results of HIV-1 subtype evaluation. In the analyzed cohort we observed the dominance of HIV-1 subtype A among individuals from eastern countries and the subtype B among people from Poland (p < 0.05).

**Table 2:** Differences in HIV-1 subtypes in Ukrainian and Polish patients

HIV-1 subtype	Patients from eastern counties (n = 61)	Polish patients (n = 56)	P
A (n/%)	48 (78.69)	11 (19.64)	< 0.001
B (n/%)	13 (21.31)	42 (75.00)	< 0.001
C (n/%)	0 (0.00)	2 (3.57)	0.227
F (n/%)	0 (0.00)	1 (1.79)	0.460

### HIV-1 Drug Resistance

Among the analyzed cohorts we assessed the presence of HIV DRMs. In the group of patients from eastern countries there were 23 (37.70%), and in the group of Polish patients 28 individuals (50.00%) with DRMs (p = 0.218), which corresponded to 43.59% of all recruited patients. Among people from eastern countries 6 patients (9.84%) and among Polish ones 16 patients (28.57%) had more than one mutation (p = 0.033). There were 3 patients from Poland (5.36%) with three drug mutations. 2 Ukrainian patients (3.45%) and 8 Polish ones (14.29%) had mutations concerning more than one class of ART (p = 0.155). Further results are shown in Table 3. We analyzed the occurrence of specific drug resistance mutations among both: patients from eastern countries and Polish patients. The comparative results are presented in Table 4.

**Table 3:** The occurrence of HIV-1 drug resistance in different classes of ART

Class of HIV drug	Mutations in patients from eastern countries (n = 23)	Mutations in Polish patients (n = 28)	P
NRTI (n/%)	5 (21.74)	4 (14.29)	0.714
NNRTI (n/%)	5 (21.74)	8 (28.57)	0.746
PI (n/%)	15 (65.22)	23 (82.14)	0.405

**Table 4:** Comparison of HIV drug resistance mutations in patients from eastern countries and Polish patients

Class of HIV drug	Drug resistance mutations	Subtype A		P	Subtype B		P
		Patients from eastern countries (n = 48)	Polish patients (n = 13)		Patients from eastern countries (n = 11)	Polish patients (n = 42)	
NRTI	M41L (n/%)	0 (0.00)	0 (0.00)	1.000	0 (0.00)	1 (2.38)	1.000
	T69N (n/%)	1 (2.08)	0 (0.00)	1.000	0 (0.00)	0 (0.00)	1.000
	T215S (n/%)	0 (0.00)	0 (0.00)	1.000	0 (0.00)	1 (2.38)	1.000
	A62V (n/%)	4 (8.33)	1 (7.69)	1.000	0 (0.00)	0 (0.00)	1.000
	K70T (n/%)	0 (0.00)	0 (0.00)	1.000	0 (0.00)	1 (2.38)	1.000
	M184V (n/%)	0 (0.00)	1 (7.69)	0.213	0 (0.00)	0 (0.00)	1.000
NNRTI	E138A (n/%)	2 (4.17)	0 (0.00)	1.000	1 (9.09)	1 (2.38)	0.375
	V106I (n/%)	0 (0.00)	1 (7.69)	0.213	0 (0.00)	5 (11.90)	0.571
	K103N (n/%)	0 (0.00)	0 (0.00)	1.000	0 (0.00)	1 (2.38)	1.000
	V179D (n/%)	0 (0.00)	0 (0.00)	1.000	0 (0.00)	1 (2.38)	1.000
	V179E (n/%)	0 (0.00)	0 (0.00)	1.000	1 (9.09)	0 (0.00)	0.208
	V90I (n/%)	1 (2.08)	0 (0.00)	1.000	0 (0.00)	0 (0.00)	1.000
PI	L10I (n/%)	8 (16.67)	1 (7.69)	0.669	2 (18.18)	10 (20.81)	1.000
	A71T (n/%)	0 (0.00)	0 (0.00)	1.000	1 (9.09)	9 (21.43)	0.667
	L10V (n/%)	6 (12.50)	0 (0.00)	0.326	0 (0.00)	5 (11.90)	0.571
	A71V (n/%)	0 (0.00)	0 (0.00)	1.000	0 (0.00)	4 (9.52)	0.569
	L33F (n/%)	1 (2.08)	0 (0.00)	1.000	0 (0.00)	0 (0.00)	1.000
	Q58E (n/%)	1 (2.08)	0 (0.00)	1.000	0 (0.00)	0 (0.00)	1.000

### Applied ART Regimen and Response to Treatment

Among the analyzed groups of patients we assessed the applied antiretroviral therapy regimens. The results are shown in Table 5. One patient from eastern countries voluntarily did not begin the treatment therefore that person was excluded from the analysis. The patients were observed from the time of diagnosis to September 2022—the mean time of observation was 20.15 months (minimum 5, maximum 52 months).

**Table 5:** Applied ART regimen in Ukrainian and Polish patients

ART regimen	Patients from eastern countries (n = 60)	Polish patients (n = 56)	P
InSTI-based (n/%)	53 (86.89)	45 (80.36)	0.346
PI-based (n/%)	5 (8.20)	7 (12.50)	0.556
NNRTI-based (n/%)	2 (3.28)	4 (7.14)	0.427

InSTI-based regimens included: bictegravir, dolutegravir, raltegravir or elvitegravir, PIs: darunavir and NNRTI-based: doravirine. There were 44 patients from eastern countries (72.13%) and 46 Polish patients (82.14%) who obtained undetectable HIV-1 viral load after 6 months of ART. Virological failure (detectable HIV-1 viral load after 6 months from the initiation of the ART), due to poor adherence (patients admitted themselves to taking their medications irregularly), was observed in 5 patients (4.27%). 4 individuals (3.42%) discontinued intentionally the treatment before 6-months since diagnosis and 3 patients (2.56%) died within 6 months from HIV diagnosis. 15 patients (12.82%) were not assessed after 6 months from the beginning of the ART due to the change of the treatment center or shorter than 6 months period since implementation of the treatment. No statistically significance was observed among the cohorts (p > 0.05).

## Discussion

### Cohort Representativeness

The number of new diagnosed HIV-1 infections in Ukraine during recent years reached amount around 16 thousand cases every year. Almost 65% of HIV-1 transmissions occur annually through heterosexual contact, 30% are due to IDU and only 3% occur among MSM [3]. If assuming that 5.6 million displaced people from Ukraine, who arrived in EU/EEA countries till May 2022, are representative for whole population of Ukraine, up to 60 thousands additional PLWH may be living in the EU/EEA. Considering the above, it’s estimated that up to 14,000 Ukrainian refugees may be living in the EU/EEA with an undiagnosed HIV infection [5, 17]. Because of the restrictions in crossing the Ukraine’s borders by men aged 18–59 years old (sexually active group and IDU), it has been estimated that among Ukrainian refugees 95.6% were juveniles and adult women. Therefore the population of Ukrainian war migrants in the EU/EEA cannot be fully representative to the whole population of this country. Despite that fact, the evaluation of molecular characteristic of the primary HIV-1 resistance to ART should not be affected by this selective border traffic, as the presence of DRMs doesn’t depend on age or sex [17–20].

In our study among ART naïve, newly diagnosed Ukrainian migrants 44% were women, the majority of patients acquired HIV infection through heterosexual contacts, which corresponds with the presented epidemiological data [3, 19]. Furthermore our study begun in 2020—before the war, while the male migrants from Ukraine to Poland were frequent. Thanks to this, the study more accurately reflects the epidemiology of infections spread, and thus HIV-1 drug resistance frequency, than a potential study limited only to the war period.

In our cohort all of the patients from Eastern Europe migrated to Poland within 6 to 12 months before the recruitment to the study, and none of them presented with acute/early retroviral infection. Migration from Ukraine to Poland, especially in recent years, including the period 2020–2022, was triggered mainly by the difficult political and economic situation (pre-war conditions). Among 58 patients from Ukraine participating in our study 11 of them (almost 20%) refugeed after beginning of the war and 47 patients had arrived to Poland earlier. Two Polish patient were excluded from the analysis because of the confirmed acquisition of HIV infection beyond the Polish borders (Holland, Ireland). Therefore in most cases our patients acquired HIV infection in the country of residence.

### Subtype A Versus B

Subtype C accounts for almost half (47%) of all HIV-1 infections, while B and A for 12% and 10%, respectively. Subtypes can combine to form a hybrid: circulating recombinant form (CRF) or unique recombinant form (URF). Both, CRF02_AG and CRF01_AE, count for 13% of the cases [21].

Subtype A remains the most prevalent strain (> 50%) in Russia, former Soviet Union countries, including Ukraine and in parts of East Africa, while subtype B predominates in western Europe (75%), Americas and Oceania. Subtype C mostly can be detected in Southern Africa and India, CRF01_AE in Asia and CRF02_AG in Western Africa. Recent studies based on near full-length genome sequencing highlights the growing importance of recombinants variants and subtype C [22, 23]. Subtype A strain of low diversity, and a new circulating recombinant form derived from it, CRF03_AB and CRF02_AG, emerged in the former Soviet Union (Ukraine) [24]. In our study, in one patient (from Kazakhstan) we detected that the 3,92% of HIV-1 population was the CRF01_AE recombinant. We haven’t performed genotyping to asses HIV sub-subtypes (including A6-a risk factor of increased virological failure to InSTI).

Because the HIV-1 highly genetic variability, there is no standard wild-type strain. Therefore, for drug-resistance studies, mutations are defined as amino acids differences from one of several wild-type reference sequences. The most commonly used reference sequences are of the laboratory virus isolates: HXB2, NL43 and a consensus reference sequence comprising the most common amino acid at each position in wild-type subtype B viruses (subtype B consensus). These sequences are almost identical, differing at a few amino acids not involved in drug resistance phenomenon. The use of subtype B consensus as reference is based on historical precedence [7, 8]. Nevertheless the data on baseline antiretroviral susceptibility derived from studies of subtype B may not be applicable to all non-B subtypes HIV. DRMs are mostly found across subtypes, but drug resistance is not well studied in non-B subtypes, CRFs and URFs. Therefore continuous surveillance for transmitted drug resistance is essential in all HIV subtypes and recombinants.

Differential characteristics of viral subtypes and their interactions with the human host may influence the ability of HIV transmission, treatment and disease progression. This concern is illustrated by HIV-2 and group O strains of HIV-1, which possess intrinsic resistance to NNRTIs [25–27]. Among others, it was also proven that likelihood of AIDS developing was increased in the patients infected with a non-A subtype, in others studies such connection was not confirmed [28–32]. In our trail 26% of Ukrainian (79% of all cohort infected with subtype A) versus 50% of Polish patients (75% of all cohort with subtype B) were late presenters (p < 0.05), which is consistent with the results from previously performed studies [28, 31, 32]. Such high prevalence of AIDS-defining diseases in our cohort (38% of all recruited patients) can be also the result of the characteristic of the sites of the recruitment: most of the centers were clinical departments, mostly the diagnosis were made because of the severe state of the patients—they sought medical care (hospitalization) due to poor general state and not due to a risky behavior in the individual anamnesis. Nevertheless the clinical state during diagnosis (but not the period of the progression to the AIDS) do not depends directly from the presence of the specific DRMs.

### Detected DRMs in the Settings of ART HIV-1 Resistance

Mutations leading to resistance appear to be similar among subtypes, but certain mutations seem to occur more frequently in non-B subtypes. Studies of HIV-1 non-B subtypes in naïve ART patients have demonstrated that protease and reverse transcriptase coding sequences vary from those of the B subtype reference strains in a predictable manner. Among others, polymorphisms in the RT gene do not occur in known sites of DRMs which corresponds to reduced sensitivity to NRTI. Data from other studies reveal that in naïve ART patients protease coding sequences from non B-subtypes contain substitutions associated with DRMs in B subtype, known to cause secondary resistance. These genotypic changes do not confer consistently decreased susceptibility by themselves when viral strains are subjected to phenotypic testing [33, 34]. Furthermore, studies of resistance pattern that emerge in patients receiving ART indicate that presence of polymorphisms before start of ART may provide a background facilitating emergence of specific selection pathways to secondary resistance[27, 35, 36].

Overall most antiretroviral resistance in non-B subtypes are covered by the current resistance databases [7, 8, 37]. Although there are potential problems with comparing responses to ART between non-B subtypes and subtype B HIV-1 patients, the available data from the clinical practice are encouraging: HIV-1 subtype did not affect response to the treatment [38–40].

As the emergence of DRMs reduce the effectiveness of antiretroviral agents, DRMs are one of the main concerns in the clinical management of individuals with HIV-1 infection. The growing body of evidence confirms that polymorphisms and primary resistance found in various subtypes before ART may affect genetic pathways of resistance. We outline considerations for developing a list of drug-resistance mutations for epidemiological estimates of transmitted resistance in the face of changing epidemiological situation in EU/EEA. The current list of mutations should not only include DRMs associated with clinical/phenotypical resistance to HIV-1 but also mutations that may contribute to a reduced virologic response to a drug, especially when occur in accumulation. Our study gives up to date information in the light of changed epidemiological situation in central and eastern Europe. In our study we haven’t recognized increased percentage of DRMs associated with clinical (phenotypical) resistance to ART, but we have detected mutations triggering reduced response to ART in almost half (44%) of the recruited patients, which support increasing concern about the emergence of HIV DRMs.

### Limitations of the Study

Because of the time of the recruitment (SARS COVID-19 epidemy) molecular diagnostic was limited—it was the major cause of lack of InSTI resistance testing. Nevertheless, it has been proven that DRMs to new generation of InSTI do not play role in the ART failure [41]. As well we haven’t performed genotyping to asses HIV sub-subtypes (including A6-a risk factor of increased virological failure to InSTI).

Additionally in the intention to treat (ITT) protocol 72–82% of patients achieved undetectable HIV viral load. But this lost to follow-up/lack of the clinical answer to ART was mainly due to short period of observation (less than 6 months) or the death of the patients. Only 3.5–5% of the patients under the observation had detectable HIV-1 viral load after 6 months from the initiation of the therapy, which is consistent with the results from RCT trails [42, 43].

Moreover, collection and analysis of sequencing data always brings along the lack of current knowledge of the association of new mutations with drug resistance, the occurrence of relevant mutations outside the regions targeted by routine resistance assays and the risk of the presence of drug-resistant minority of HIV virus population.

### New Contribution to the Literature

From a humanitarian and public health perspective, it is vital that migrants living with HIV and/or at risk for HIV who are displaced from Ukraine have access to HIV care services in EU/EEA. We have demonstrated that the HIV-1 subtype pattern in refugees from eastern countries is different: among individuals from Ukraine: HIV-1 subtype A was the dominant strain. DRMs were found in almost half of all the cases, different mutations in the group of patients from eastern countries and in Polish patients were detected, but not with statistical significance. The results from our trial show that Ukrainian migrants from our cohort do not carry elevated number of HIV-1 DRMs and increased risk of transmission with HIV multidrug resistant strains. Furthermore high prevalence of DRMs in our study confirms increasing concern about the emergence of HIV drug resistance mutations triggering reduced response to ART, which can lead to treatment failure.
